# Intervertebral Disk Degeneration and Bone Mineral Density: A Bidirectional Mendelian Randomization Study

**DOI:** 10.1007/s00223-023-01165-1

**Published:** 2023-11-17

**Authors:** Jie Zhao, Jingyu Wang, Haixu Xu, Wei Hu, Fangyuan Shi, Zhengrui Fan, Chunlei Zhou, Hong Mu

**Affiliations:** 1 Department of Clinical Lab, Tianjin First Central Hospital, 300192 Tianjin, China; 2 Department of Orthopedics, Tianjin University Tianjin Hospital, 300211 Tianjin, China; 3 Department of Immunology, School of Basic Medical Sciences, Tianjin Medical University, 300070 Tianjin, China; 4 Department of Spine Surgery, Tianjin People’s Hospital, 300122 Tianjin, China; 5 School of Information Engineering, Ningxia University, Yinchuan, China

**Keywords:** Osteoporosis, Intervertebral disk degeneration, Bone mineral density, Genome-wide association study, Mendelian randomization

## Abstract

This study aimed to investigate the causal relationship between bone mineral density (BMD) and intervertebral disk degeneration (IVDD) using a two-sample bidirectional Mendelian randomization analysis. Summary-level data from the Genome-Wide Association Study (GWAS) were used. Instrumental variables (IVs) for IVDD were selected from the large-scale Genome-Wide Association Study (GWAS) (20,001 cases and 164,682 controls). Bone mineral density (BMD) at five different sites (heel (*n* = 426,824), total body (TB) (*n* = 56,284), forearm (FA) (*n* = 8143), femoral neck (FN) (*n* = 32,735), and lumbar spine (LS) (*n* = 28,498)) was used as a phenotype for OP. Bidirectional causality between IVDD and BMD was assessed using inverse variance weighting (IVW) and other methods. Related sensitivity analyses were performed. Myopia was also analyzed as a negative control result to ensure the validity of IVs. Heel bone mineral density (heel BMD), total body bone mineral density (TB-BMD), femoral neck bone mineral density (FN-BMD), and lumbar spine bone mineral density (LS-BMD) have a direct causal relationship on intervertebral disk degeneration (IVDD) [heel BMD-related analysis: beta = 0.06, *p* = 0.03; TB-BMD-related analysis: beta = 0.18, *p* = 8.72E-08; FN-BMD-related analysis: beta = 0.15, *p* = 4.89E-03; LS-BMD-related analysis: beta = 0.16, *p* = 1.43E-04]. There was no evidence of a significant causal effect of IVDD on BMD. In conclusion, our study found a significant positive causal effect of lower BMD on IVDD, and we identified significant causal effects of heel, TB-, FN-, and LS-BMD on IVDD, but there was no evidence of a significant causal effect of IVDD on BMD.

**Supplementary Information:**

The online version contains supplementary material available at 10.1007/s00223-023-01165-1.

## Introduction

Intervertebral disk degeneration (IVDD) is a common degenerative disease characterized by a progressive decrease in the proteoglycan and water content of the nucleus pulposus (NP), an age-dependent, cell-mediated molecular process that may eventually lead to rupture of the disks between the vertebrae [1, 2].Degenerated disks are more likely to herniate and may compress spinal nerves and nerve roots. Although asymptomatic in some cases, disk degeneration is known to be associated with herniated or prolapsed disks, low back pain and sciatica [3, 4]. As an increasingly prevalent health problem, IVDD significantly affects the quality of life of patients and imposes a heavy economic burden on countries with rapidly aging populations, such as China [5].The etiology and pathophysiology of IVDD have not been fully characterized. It may be the result of a combination of genetic background and environmental factors, including aging, overload, physical activity, and smoking [6, 7].

Osteoporosis (OP), like intervertebral disk degeneration (IVDD), is a common age-related condition that frequently causes systemic bone disease consisting of decreased bone density and mass, destruction of bone microarchitecture, and increased bone fragility [8]. Bone mineral density (BMD) is a highly heritable trait and an important indicator of bone strength, and the diagnosis of osteoporosis in clinical and epidemiologic studies is based on BMD, which is usually measured by dual-energy X-ray absorptiometry [9]. Both IVDD and OP exhibit strong genetic components. Relevant studies have found a heritability rate of 34–61% for IVDD at various spinal sites [10], while the heritability of OP reaches as high as 50–85% [11]. This suggests the existence of shared genetic mechanisms and biological processes between these two conditions. For instance, inflammatory cytokines, including TNF-α, IL-17, IL-6, and IL-1, play crucial roles in the onset and progression of OP [12], and they are similarly closely associated with the development of IVDD [13]. Furthermore, numerous observational studies have also indicated a close relationship between OP and IVDD [14]. Naohisa Miyakoshi et al. [15] investigated the relationship between bone mineral density in the lumbar spine (anterior–posterior, lateral, and medial–lateral) and proximal femur (femoral neck, trochanter, and Ward’s triangle) and intervertebral disk degeneration in postmenopausal women, proving that osteoporosis is negatively correlated with intervertebral disk degeneration. Tobias A Mattei et al. [16] found that osteoporosis can delay disk degeneration by increasing diffusive transport of nutrients through mechanical and vascular pathophysiologic pathways within the intervertebral disks. A study by Harada et al. [17] and another by Xiangwen Li et al. [18] present a contrasting viewpoint, suggesting that osteoporosis promotes the development of IVDD; however, most of these studies were cohort or cross-sectional studies, and it was not possible to draw conclusions about causality. In addition, there are also studies that provide conflicting evidence against such an association [14].

Does low/high BMD have a direct effect on IVDD and vice versa? Due to confounding factors, these prior observational data are limited to inferences of association. The randomized controlled trial (RCT) design is the gold standard method for determining causality; however, it can be time and money consuming. The Mendelian randomization (MR) is a method that uses single-nucleotide polymorphisms (SNPs) as instrumental variables (IVs) to assess the causal effect of an exposure on an outcome and is now widely used to assess the causal impact of risk factors on an outcome [19]. As it is randomly assigned due to genetic variation, adverse effects due to potential confounders or reverse causality can be minimized [20]. To the authors’ knowledge, MR between IVDD and BMD has not been investigated, while no randomized controlled trials (RCTs) have directly assessed bidirectional relationships. Therefore, we performed a bidirectional two-sample MR analysis to explore the causal relationship between IVDD and BMD.

## Materials and Methods

### Study Design

**Fig. 1:**
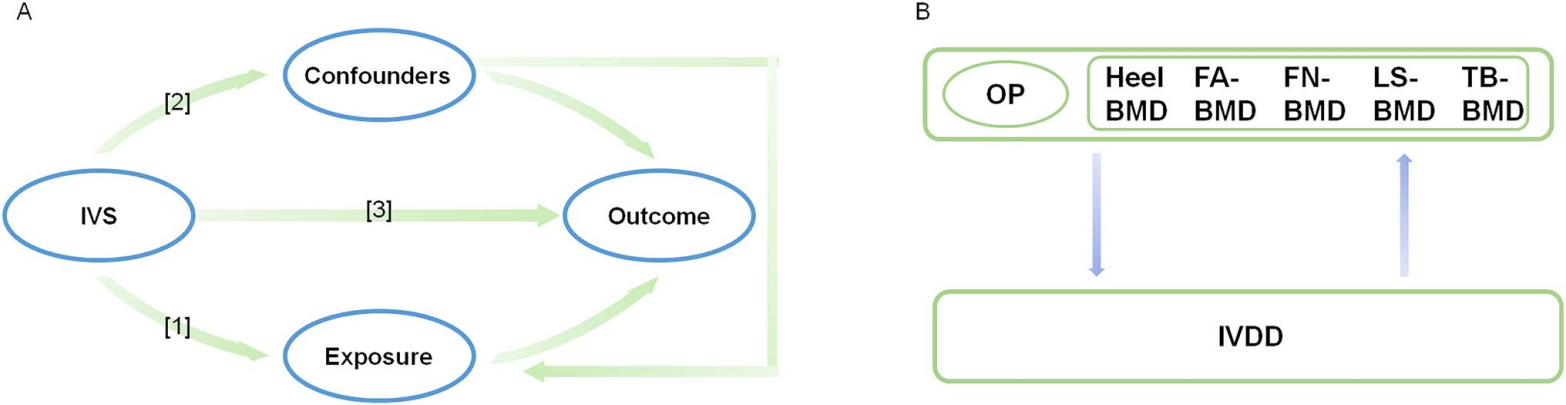
Schematic diagram of assumptions and process analysis for bidirectional MR analysis **A** [1] the genetic IVs used are strongly associated with exposure; [2] the selected IVs are not associated with potential confounders; and [3] the IVs can only influence the outcome risk through exposure. **B** Steps in bidirectional MR analysis: osteoporosis was used as an exposure in the first step, while IVDD was studied as an outcome, and vice versa in the second step. The arrows represent the direction of the causal relationship between the two of the results. *IVs* instrumental variables, *OP* osteoporosis, *IVDD* intervertebral disk degeneration, *TB* total body, *FA* forearm, *FN* femoral neck, *LS* lumbar spine, *BMD* bone mineral density, *MR* Mendelian randomization

The flow of the experimental study design is shown in Fig. 1. The selected instrumental variables (IVs) valid for MR analysis need to be base d on three assumptions: (1) the genetic IVs used are strongly associated with exposure; (2) the selected IVs are not associated with potential confounders; and (3) the IVs can only influence the outcome risk through exposure. The current study used a bidirectional MR analysis in two steps: the OP was studied as an exposure and the IVDD was studied as an outcome in the first step, while the opposite was true in the second.

### Data Sources

The GWAS summary of IVDD data from the FinnGen consortium includes a total of 20,001 cases and 164,682 controls from European populations [21]. IVDD was diagnosed by ICD-10 M51, ICD-9 722 and ICD-8 275 and excluded by ICD-9 7220\7224\7227\7228A, ICD-8 7250. Genetic values were also corrected for sex, age, 10 PCs, genotyping batch for all individuals of European ancestry. The GWAS summary statistics for osteoporosis (heel, TB-, FA-, FN- and LS-BMD) used for analysis in this study were obtained from GEFOS (http://www.gefos.org/). The GWAS of heel BMD comprised 426,824 individuals from the European population in the UK Biobank study. The GWAS on heel BMD was quantitatively estimated by heel ultrasound, and age, sex, genotyping array, assessment center, and ancestry information principal components 1–20 were incorporated as covariates in a fixed model [22]. The GWAS dataset for TB-BMD contains summary statistics from GWAS meta-analysis studies involving 56,284 European individuals, adjusted for age, weight, height, sex, genomic principal components, and other study-specific covariates (e.g., recruitment centers) [23].Associated GWAS summary statistics for FA- (*n* = 8143), FN- (*n* = 32,735), and LS-BMD (*n* = 28,498) were obtained from the Genetic Factors in Osteoporosis (GEFOS) Consortium (http://www.gefos.org/) [24]. Genetic values were corrected and standardized for sex, age, age squared, and weight for the 69,376 individuals of European ancestry in the data.

### Genetic Instrumental Variable Selection

Independent single-nucleotide polymorphisms (SNPs) across the genome that were strongly correlated (*p* < 5 × 10^–8^) with exposure were selected as instrumental SNPs (Linkage disequilibrium, clumping *r*^2^ = 0.001 and kb = 10,000) based on the three hypotheses of the MR analysis [25]. We correlate the corresponding SNPS with the outcome. For SNPs that were not feasible in the results, we utilized proxy SNPs that were highly correlated (*r*^2^ > 0.8) with the requested SNPs. To guarantee alignment of all risk factors and outcome alleles on the same strand, we harmonized the effects of these instrumental SNPs whenever feasible. To further verify the strength of the selected IVs, we calculated the F-statistics of the selected IVs using an online tool (https://sb452.shinyapps.io/overlap) [19]. Genetic IVs with F-statistics > 10 demonstrate the good strength of the tool to mitigate potential bias in MR analysis.

### MR Analysis

The causal effect between exposure and outcome was assessed by the IVW method, which is calculated as the effect size of the association between SNP-outcome divided by the effect size of the association between SNP-exposure [26]. The IVW method is recognized as the most precise approach for estimating causality when the data are shown to be free of directional pleiotropy (*p* > 0.05 for the MR-Egger intercept) [27]. Meanwhile, the random-effects model was applied to the analysis of instrumental variables without evidence of heterogeneity (*p* > 0.05 for MR heterogeneity), otherwise the fixed-effects model was used for the analysis. The causal effect of exposure on outcome was calculated using MR-Egger, weighted median, and weighted mode methods to further ensure the robustness of the experimental results [20, 28].For IVs with *p*-values < 0.05 in IVW analyses, we subsequently performed MR-PRESSO using the MR-PRESSO package, which detects, removes, and provides outlier-adjusted estimates for potentially pleiotropic IVs (outliers) [29]. All data were analyzed by R Statistical Software (version 4.3.0) and the R packages “TwosampleMR,” “MendelianRandomization,” and “MR-PRESSO.”

### Sensitivity Analysis

To ensure the robustness of the results of the final MR analyses, multinomial sensitivity analyses were conducted. Cochran’s Q statistic was used to analyze potential heterogeneity among the IVs. Horizontal pleiotropy of IVs was then tested by MR-Egger regression, which was judged by the p-value of the intercept term; if *p* < 0.05, the IVs were shown to be horizontally pleiotropic, and vice versa, they were not. Finally, leave-one-out analysis was used to test whether chance associations were driven by a single SNP.

### Negative Control

Myopia was used as a negative control result to ensure the validity of IVs as there is no evidence of an association between myopia and IVDD or OP. GWAS data for myopia were obtained from the FinnGen Biobank (https://www.finngen.fi/en) and included 37,362 cases of myopia and 423,174 controls from European populations.

## Results

### The Influence of Osteoporosis on Intervertebral Disk Degeneration

Overall, we obtained 4,948,032,122 IVs that were not associated with linkage disequilibrium (*r*^2^ < 0.001) and reached a genome-wide significance level (*p* < 5 × 10^–8^) in GWAS from heel, TB-, FA-, FN-, and LS-BMD, respectively (Supplementary Table 1). Meanwhile, the F-statistics of all IVs were statistically greater than 10, implying that the selected IVs were robust enough to eliminate potential bias. As shown in Table 1, except for the IVs in FN-, LS-BMD, the selected IVs in heel, TB-, and FA-BMD showed significant heterogeneity (*p* < 0.05) in the heterogeneity test, so a random-effects model was used in the computation of inverse variance weight (IVW). According to the intercept of the MR-Egger regression, there was no horizontal pleiotropy between all exposures and outcomes (Table 1, *p* > 0.05). Leave-one-out sensitivity analyses confirmed that no single SNP was found to have a large effect on the overall outcome in the five groups of heel, TB-, FA-, FN-, and LS-BMD (Supplementary Figs. 1–5). Negative control results indicated that heel BMD, TB-BMD, FA-BMD, FN-BMD, and LS-BMD were not associated with myopia, suggesting that the exposure IVs selected for this study were appropriate (Supplementary Tables 2 and 3).

**Table 1:** Mendelian randomization estimates for BMD on IVDD

Exposure	Outcome	No. of Ivs	Heterogeneity tests		Directional horizontal pleiotropy test	MR results		
			Methods	Cochran’s Q (p)	MR-Egger intercept (p)	Method	Beta	*P*
Heel BMD	IVDD	494	MR Egger	822.36 (5.21E-19)	− 8.89E-04 (0.53)	Inverse variance weighted	0.06	**0.03**
			Inverse variance weighted	823.01 (5.93E-19)		MR Egger	0.09	0.08
						Weighted median	0.04	0.32
						Weighted mode	0.05	0.20
						MR Presso	0.06	**0.03**
TB BMD	IVDD	80	MR Egger	110.65 (8.88E-03)	6.84E-03 (0.17)	Inverse variance weighted	0.18	**8.72E-08**
			Inverse variance weighted	113.37 (6.82E-03)		MR Egger	0.07	**4.66E-01**
						Weighted median	0.17	**4.82E-04**
						Weighted mode	0.15	**5.74E-02**
						MR Presso	0.15	**8.88E-06**
FA BMD	IVDD	3	MR Egger	9.96 (1.60E-03)	− 1.58E-02 (0.86)	Inverse variance weighted	0.10	0.43
			Inverse variance weighted	10.49 (5.27E-03)		MR Egger	0.22	0.75
						Weighted median	0.15	**0.04**
						Weighted mode	0.17	0.15
FN BMD	IVDD	21	MR Egger	22.83 (2.45E-01)	− 2.79E-02 (0.10)	Inverse variance weighted	0.15	**4.89E-03**
			Inverse variance weighted	26.54 (1.49E-01)		MR Egger	0.59	**0.03**
						Weighted median	0.12	0.08
						Weighted mode	0.05	0.76
						MR Presso	0.15	**0.01**
LS BMD	IVDD	22	MR Egger	20.65 (4.18E-01)	1.59E-02 (0.16)	Inverse variance weighted	0.16	**1.43E-04**
			Inverse variance weighted	22.90 (3.49E-01)		MR Egger	− 0.05	0.76
						Weighted median	0.20	**1.01E-03**
						Weighted mode	0.24	**0.04**
						MR Presso	0.13	**8.59E-03**

Bold values indicate statistically significant at *P* < 0.05

*BMD* bone mineral density, *IVDD* intervertebral disk degeneration, *FA* forearm, *FN* femoral neck, *LS* lumbar spine, *TB* total body, *IVs* instrumental variables

The results of this MR study are mainly correlated with the IVW analysis. As shown in Table 1, the results of IVW support the existence of a causal relationship between heel, TB-, FN-, and LS-BMD on IVDD (heel BMD-related analysis: beta = 0.06, *p* = 0.03; TB-BMD-related analysis: beta = 0.18, *p* = 8.72E-08; FN-BMD-related analysis: beta = 0.15, *p* = 4.89E-03; LS-BMD-related analysis: beta = 0.16, *p* = 1.43E-04;). Meanwhile, its beta values implied that heel, TB-, FN-, and LS-BMD were all positively correlated with IVDD. MR Pleiotropy RESidual Sum and Outlier (MR-PRESSO) detected several potentially pleiotropic IVs in BMD, which were rs7814941 (heel BMD), rs4757350 (TB-BMD). After removing outliers, the causal relationship between BMD and outcome remained significant. Based on the results of different MR methods, we concluded that there is a causal relationship between BMD on IVDD, which means that people with low BMD are more likely to develop IVDD.

### The Influence of Intervertebral Disk Degeneration on Osteoporosis

In the second stage, the effect of disk degeneration on osteoporosis was investigated. We screened six IVs with genome-wide significance levels (*p* < 5 × 10^–8^) of LD-independent (*r*^2^ < 0.001) from the GWASs for IVDD (Supplementary Table 4). The F-statistics of the IVs for the selected IVDDs were all greater than 10 to ensure that the selected IVs were sufficiently robust enough to eliminate the potential bias. As shown in Table 2, the heterogeneity test indicated that none of the selected IVs in the groups expressed significant heterogeneity (*p* < 0.05), except for the IVs of the selected IVDDs on the heel BMD. MR-Egger regression test results confirmed that none of the IVs were horizontally pleiotropic (p for MR-Egger intercept > 0.05) and all tests for leave-one-out analysis were negative (Supplementary Figs. 6–10). Meanwhile, no outlier IVs were found in the MR-PRESSO analysis. Negative control analyses showed that IVDD was not associated with myopia, suggesting that our choice of exposure IVs in this study was appropriate (Supplementary Tables 2 and 3).

**Table 2:** Mendelian randomization estimates for IVDD on BMD

Exposure	Outcome	No. of Ivs	Heterogeneity tests		Directional horizontal pleiotropy test	MR results		
			Methods	Cochran’s Q (p)	MR-Egger intercept (p)	Method	Beta	*P*
IVDD	Heel BMD	6	MR Egger	32.53 (1.49E-06)	0.03 (0.14)	Inverse variance weighted	− 0.06	0.11
			Inverse variance weighted	60.28 (1.06E-11)		MR Egger	− 0.50	0.11
						Weighted median	− 0.04	0.01
						Weighted mode	− 0.04	0.07
IVDD	TB BMD	6	MR Egger	4.41 (0.35)	0.05 (0.06)	Inverse variance weighted	− 0.03	0.55
			Inverse variance weighted	11.51 (0.04)		MR Egger	− 0.73	0.06
						Weighted median	− 0.04	0.40
						Weighted mode	− 0.04	0.52
IVDD	FA BMD	6	MR Egger	2.17 (0.70)	0.05 (0.43)	Inverse variance weighted	− 0.08	0.39
			Inverse variance weighted	2.93 (0.71)		MR Egger	− 0.71	0.39
						Weighted median	− 0.06	0.62
						Weighted mode	− 0.05	0.76
IVDD	FN BMD	6	MR Egger	2.81 (0.59)	0.02 (0.49)	Inverse variance weighted	− 0.04	0.38
			Inverse variance weighted	3.40 (0.64)		MR Egger	− 0.31	0.43
						Weighted median	− 0.06	0.27
						Weighted mode	− 0.10	0.33
IVDD	LS BMD	6	MR Egger	7.31 (0.12)	− 1.57E-03 (0.97)	Inverse variance weighted	0.09	0.17
			Inverse variance weighted	7.31 (0.20)		MR Egger	0.11	0.86
						Weighted median	0.06	0.35
						Weighted mode	0.06	0.66

*BMD* bone mineral density, *IVDD* intervertebral disk degeneration, *FA* forearm, *FN* femoral neck, *LS* lumbar spine, *TB* total body, *IVs* instrumental variables

As shown in Table 2, the IVW results indicated no causal effect of IVDD on BMD at any site (IVDD (heel BMD)-related analysis: beta = − 0.06, *p* = 0.11; IVDD (TB-BMD)-related analysis: beta = − 0.03, *p* = 0.55; IVDD (FA-BMD) -related analysis: beta = − 0.08, *p* = 0.39; IVDD (FN-BMD)-related analysis: beta = − 0.04, *p* = 0.38; IVDD (LS-BMD)-related analysis. beta = 0.09, *p* = 0.17). Meanwhile, the analysis of the results of MR-Egger, weighted median, and weighted mode methods confirmed that there was no significant causal effect of IVDD on heel, TB-, FA-, FN-, and LS-BMD. When *p* > 0.05 for the MR-Egger intercept, we considered the IVW method to be the most reliable for MR analysis. Thus, the results of the different MR methods support the conclusion that IVDD has no causal effect on BMD.

## Discussion

In summary, the present study used bidirectional two-sample MR analysis to explore whether BMD has a potential causal relationship with IVDD or vice versa. Based on the results of the MR analyses in this study, we successfully demonstrated a significant positive causal effect of heel, TB-, FN-, and LS-BMD on IVDD, i.e., people with low BMD were more likely to develop IVDD; however, there was no evidence of a significant causal effect of IVDD on BMD. To the best of our knowledge, this is the first bidirectional two-sample MR study addressing the causal relationship between BMD and IVDD that takes into account potential confounders.

Currently, several studies have suggested a possible association between OP and IVDD, but there is no uniform conclusion. The early view was that a decrease in BMD would bring about progressive endplate degeneration and thus exacerbate disk degeneration [15, 16, 30]. However, studies of the relationship between osteoporosis and disk degeneration in pre and postmenopausal women have found that patients with lower BMD appear to have a lower incidence of IVDD [15, 17, 18, 31]. Liang et al. [32] measured cervical HU values in 324 patients with degenerative cervical spondylosis and found that the HU values of cervical vertebral segments were inconsistent and unevenly distributed, and that decreased vertebral BMD and vertebral osteoporosis may trigger or exacerbate adjacent disk degeneration. Fujita et al. [33] analyzed the clinical data from musculoskeletal examination of 276 subjects and examined the independent correlation between osteoporosis and IVDD at the levels of L3/4, L4/5, and L5/S. The results showed that osteoporosis was negatively correlated with IVDD, suggesting that when bone density is maintained at higher levels, IVD degeneration may progress more rapidly. Meanwhile, Kague et al. [14] found that both too high and too low BMD were associated with premature development of IVDD on a constructed model of IVDD in adult zebrafish, rejecting the theory that low BMD is a protective factor for IVDD.

However, there is still no conclusive evidence as to whether lower BMD has a causal effect on IVDD or vice versa. There are various theories for explaining the correlation between BMD and IVDD. Since there are almost no blood vessels in the intervertebral disks and only a few tiny capillaries exist in the outer annulus of the vertebral body, the nutrient supply of the intervertebral disks mainly comes from the transportation of the upper and lower cartilage endplates. Related studies have shown that higher BMD increases the static compressive force of the endplates, which hinders the diffusion of nutrients, such as glucose, to the disk level, thus promoting IDD [34, 35]. Lower BMD, on the other hand, increases the diffusive transport of nutrients within the intervertebral disk via mechanical and vascular pathophysiological pathways, thereby slowing disk degeneration [16]. Margulies et al. [36] in their measurement of bone density in the lumbar spine found that decreased bone density reduces the number of vertebral trabeculae, increases bone fragility, and leads to microfractures under the upper endplates of the lumbar spine, which affects the nutrient supply to the lumbar spine and upper disks and ultimately promotes IVDD. In a study of IVDD using ovariectomized rhesus monkeys, Zhong et al. [37] found that osteoporosis exacerbated IVDD by increasing calcification of the endplates near the degenerating disks and decreasing nutrient vascularization. Homminga et al. [38] suggested that lumbar disk degeneration with degeneration of the nucleus pulposus, fibrosis of the nucleus pulposus, calcification of the cartilaginous endplates, and formation of peripheral osteophytes leads to a shift in loading from the internal nucleus pulposus to the annulus fibrosus, decreasing the density of the core of the trabeculae while increasing the density of the vertebral cortex.

To the best of our knowledge, no studies have been reported on the MR associated with the effects of IVDD on BMD or BMD on IVDD; therefore, this study investigated the causal relationship between IVDD and BMD using a bidirectional MR study. The design of this study facilitated robust confounding and reversal of causality and clearly elucidated the causal relationship between the relevant features of low BMD and IVDD. Also, several different MR analysis methods were used to ensure the correctness and validity of the results, and sensitivity analyses such as MR-PRESSO were performed to obtain consistent estimates of the magnitude of the causal effect of MR, and largely consistent results were obtained. Additionally, features irrelevant to IVDD and BMD (myopia) were added as additional negative controls to ensure the validity of the selected IVs and to further validate the results of the analysis. However, some potential limitations of this study remain. First, the prevalence of OP and IVDD varied by age and sex, but the present study was analyzed based on data at the pooled level of GWAS, which did not allow for subgroups to assess the impact according to different ages and sexes; second, the results of the present study showed a significant causal relationship between heel, TB-, FN-, and LS-BMD and IVDD, but FA-BMD had no significant causality, so further MR studies with larger sample sizes or randomized controlled experiments are needed, and finally, although the present study performed multiplicity test and MR-PRESSO to eliminate as much as possible the confounding caused by multiplicity, the residual bias of MR technique is an unavoidable drawback.

## Conclusions

In conclusion, our study found a significant positive causal effect of lower BMD on IVDD, and we identified a significant causal effect of heel, TB-, FN-, and LS-BMD on IVDD, but there was no evidence of a significant causal effect of IVDD on BMD. Based on our current results, BMD measurement is recommended in patients with IVDD.

### Supplementary Information

Below is the link to the electronic supplementary material.

Supplementary Figure 1 MR leave-one-out sensitivity analysis for ‘heel-BMD’ on ‘IVDD’

Supplementary Figure 2 MR leave-one-out sensitivity analysis for ‘TB-BMD’ on ‘IVDD’

Supplementary Figure 3 MR leave-one-out sensitivity analysis for ‘FA-BMD’ on ‘IVDD’

Supplementary Figure 4 MR leave-one-out sensitivity analysis for ‘FN-BMD’ on ‘IVDD’

Supplementary Figure 5 MR leave-one-out sensitivity analysis for ‘LS-BMD’ on ‘IVDD’

Supplementary Figure 6 MR leave-one-out sensitivity analysis for ‘IVDD’ on ‘heel-BMD’

Supplementary Figure 7 MR leave-one-out sensitivity analysis for ‘IVDD’ on ‘TB-BMD’

Supplementary Figure 8 MR leave-one-out sensitivity analysis for ‘IVDD’ on ‘FA-BMD’

Supplementary Figure 9 MR leave-one-out sensitivity analysis for ‘IVDD’ on ‘FN-BMD’

Supplementary Figure 10 MR leave-one-out sensitivity analysis for ‘IVDD’ on ‘LS-BMD’

Supplementary file11 (XLSX 49 KB)

Supplementary file12 (XLSX 11 KB)

Supplementary file13 (XLSX 49 KB)

Supplementary file14 (XLSX 12 KB)

## Data Availability

The original contributions presented in the study are included in the article/ Supplementary Materials. Further inquiries can be directed to the corresponding author.

## Abbreviations

GWAS: Genome-wide association studies
IVs: Instrumental variables
OP: Osteoporosis
IVDD: Intervertebral disk degeneration
TB: Total body
FA: Forearm
FN: Femoral neck
LS: Lumbar spine
BMD: Bone mineral density
MR: Mendelian randomization
SE: Standard error
SNP: Single-nucleotide polymorphism
LD: Linkage disequilibrium
IVW: Inverse variance weighted
