# Supplementary figures and images for: Intervertebral Disk Degeneration and Bone Mineral Density: A Bidirectional Mendelian Randomization Study
Source: Calcif Tissue Int. 2023 Nov 17;114(3):228–36. doi: 10.1007/s00223-023-01165-1 (PMC10902056; doi:10.1007/s00223-023-01165-1)

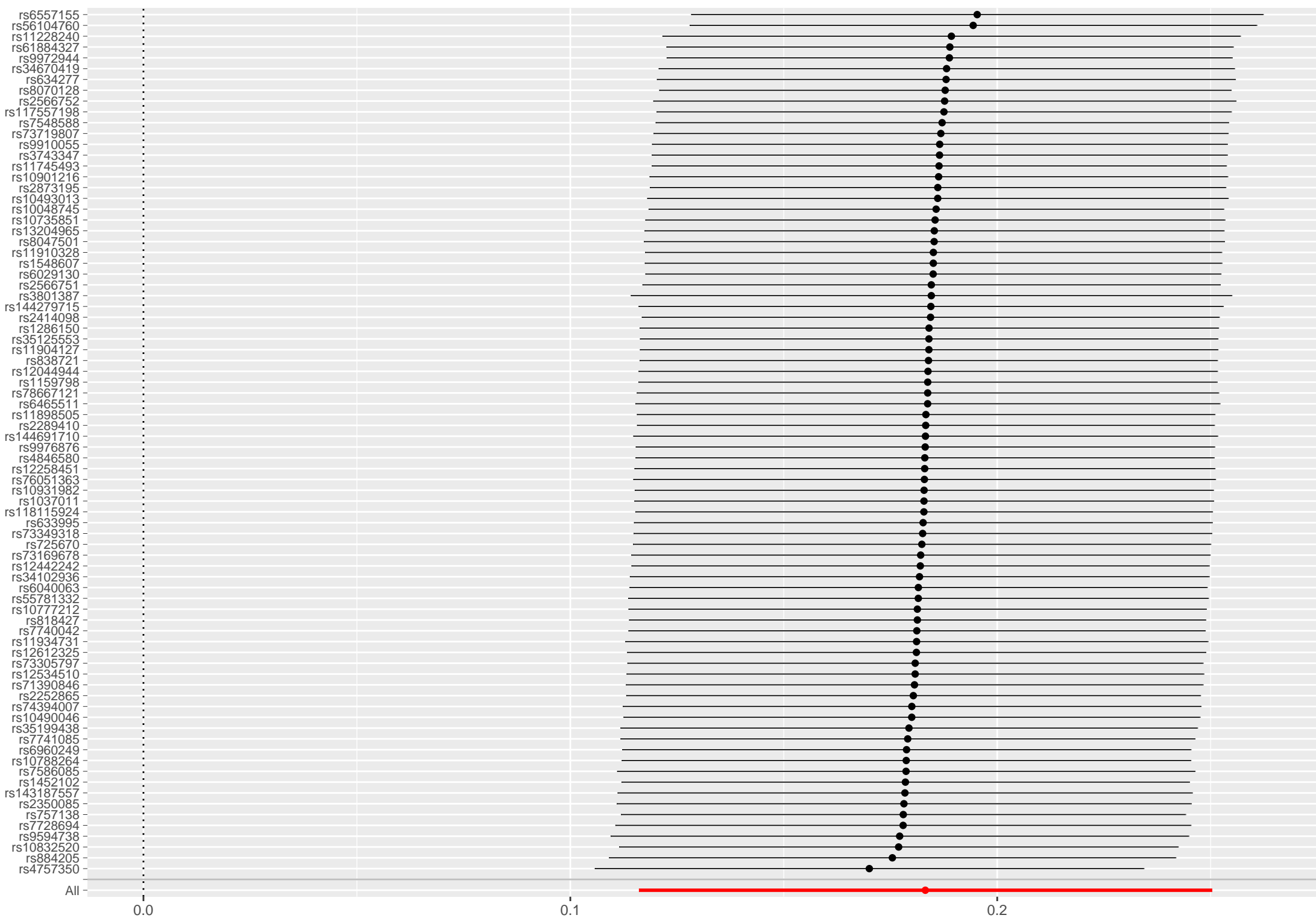

Supplement: Supplementary file 2 — Supplementary Figure 2 MR leave-one-out sensitivity analysis for ‘TB-BMD’ on ‘IVDD’ [file 223_2023_1165_MOESM2_ESM.pdf]

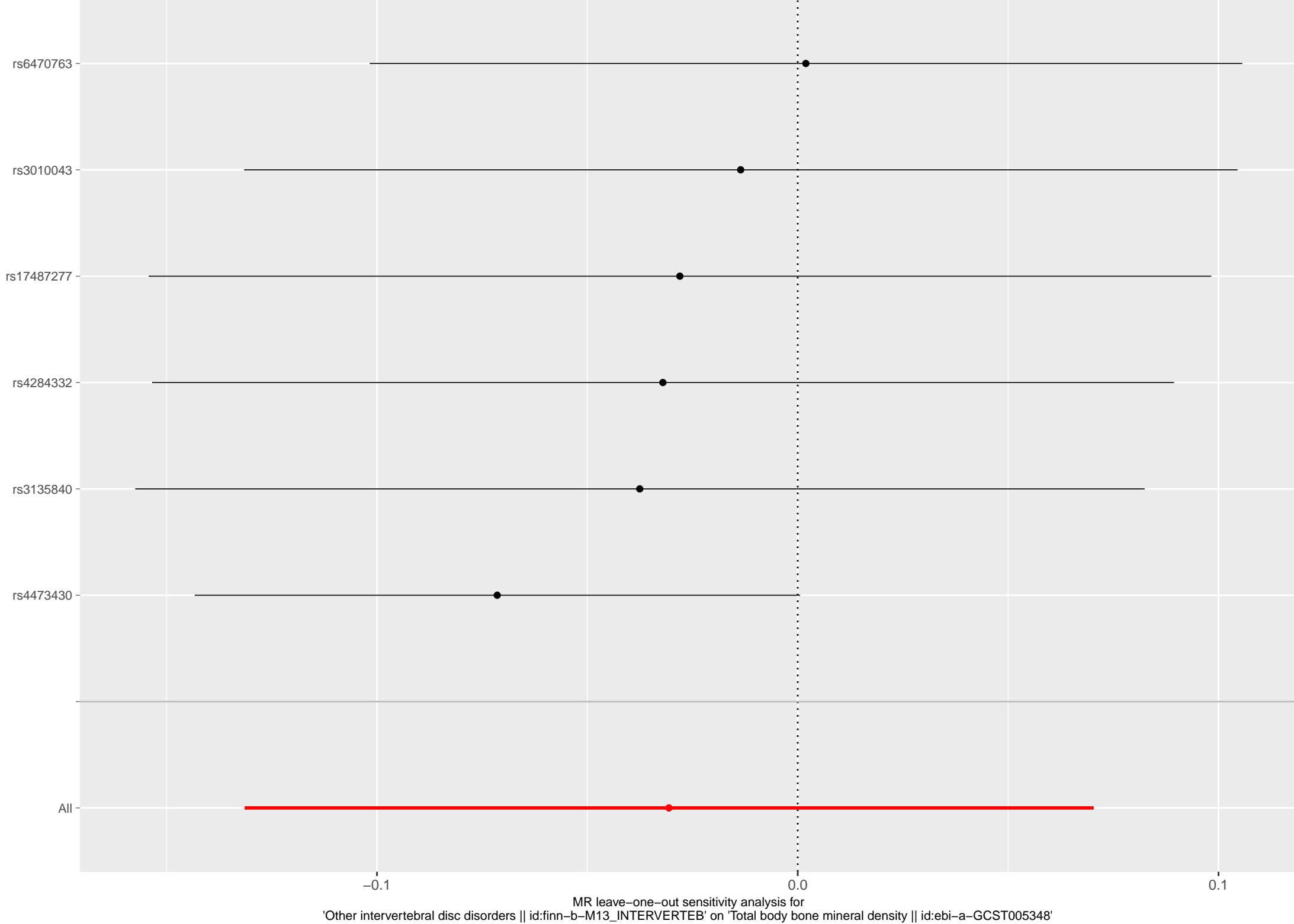

Supplement: Supplementary file 7 — Supplementary Figure 7 MR leave-one-out sensitivity analysis for ‘IVDD’ on ‘TB-BMD’ [file 223_2023_1165_MOESM7_ESM.pdf]

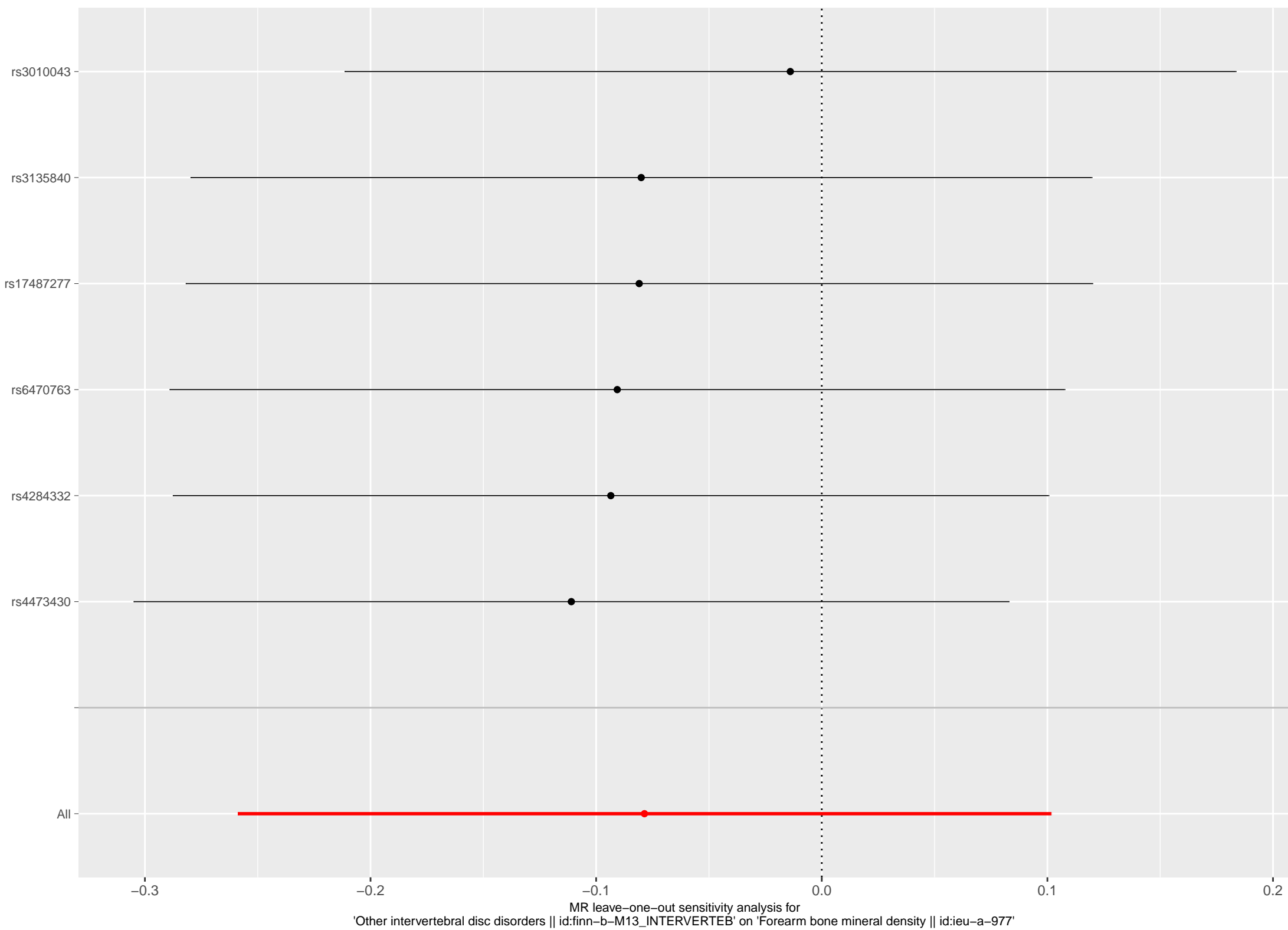

Supplement: Supplementary file 8 — Supplementary Figure 8 MR leave-one-out sensitivity analysis for ‘IVDD’ on ‘FA-BMD’ [file 223_2023_1165_MOESM8_ESM.pdf]

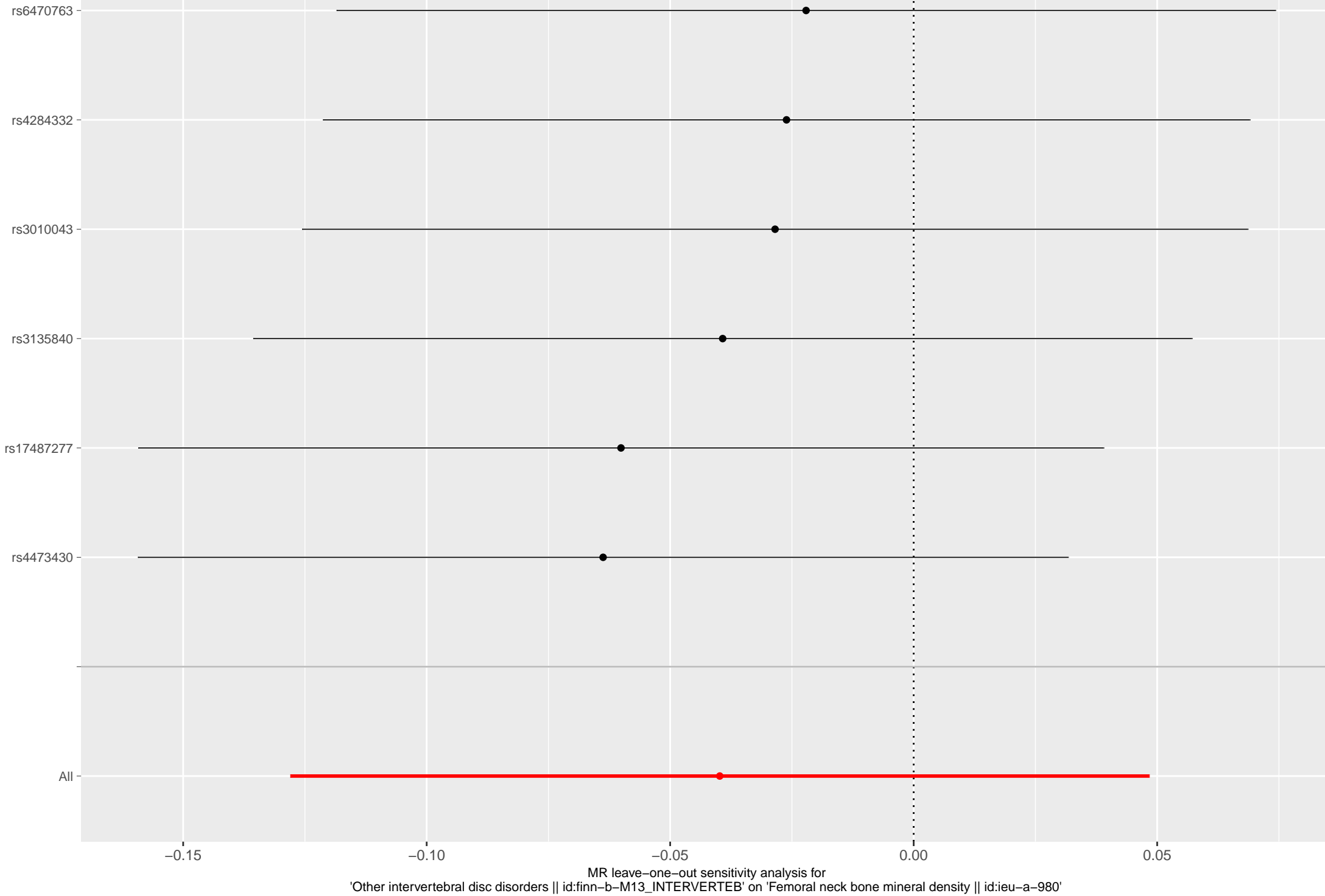

Supplement: Supplementary file 9 — Supplementary Figure 9 MR leave-one-out sensitivity analysis for ‘IVDD’ on ‘FN-BMD’ [file 223_2023_1165_MOESM9_ESM.pdf]

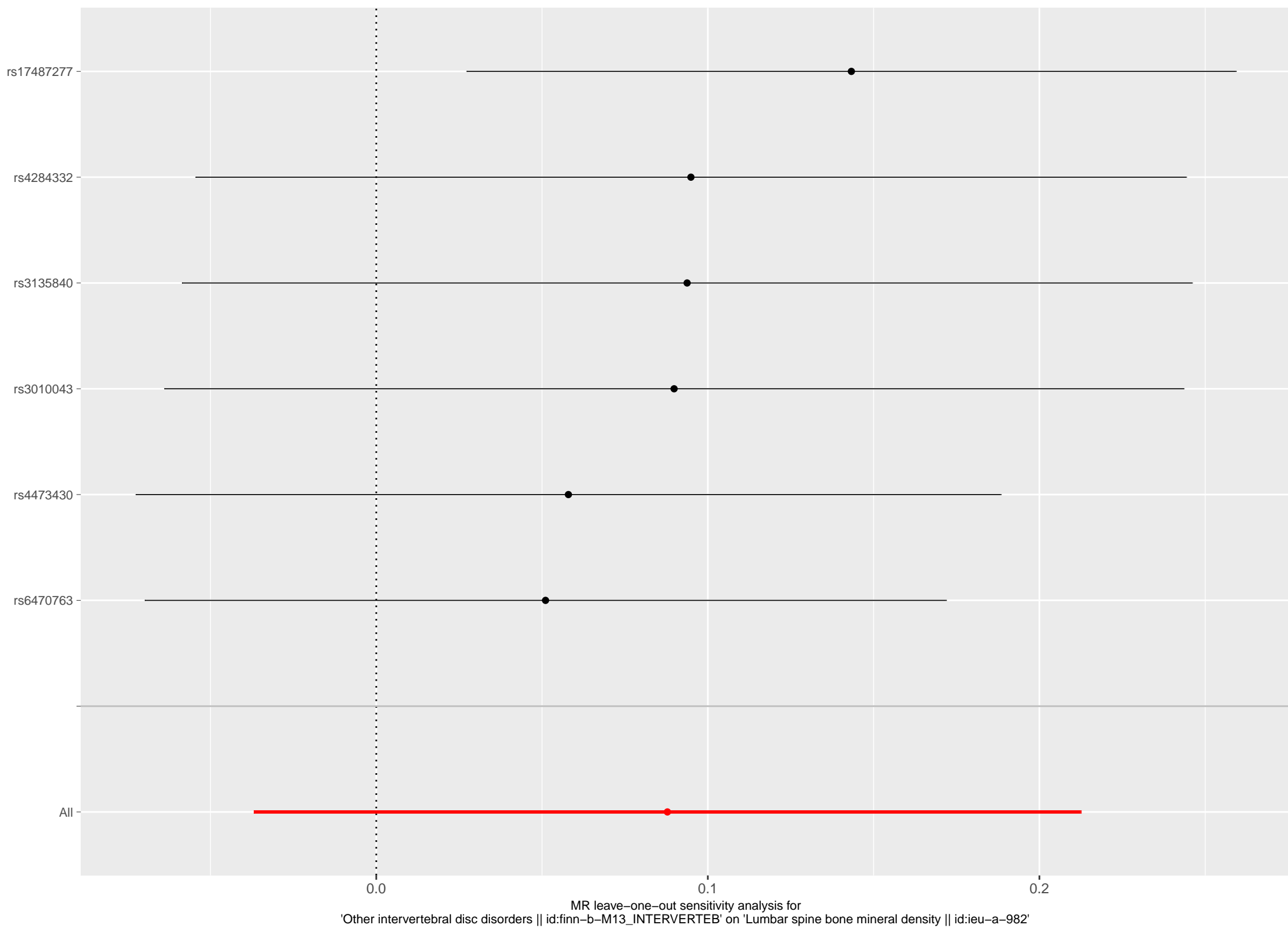

Supplement: Supplementary file 10 — Supplementary Figure 10 MR leave-one-out sensitivity analysis for ‘IVDD’ on ‘LS-BMD’ [file 223_2023_1165_MOESM10_ESM.pdf]
