# Supplementary material for: Intervertebral Disk Degeneration and Bone Mineral Density: A Bidirectional Mendelian Randomization Study
Source: Calcif Tissue Int. 2023 Nov 17;114(3):228–36. doi: 10.1007/s00223-023-01165-1 (PMC10902056; doi:10.1007/s00223-023-01165-1)
Supplement: Supplementary file 4 — Supplementary Figure 4 MR leave-one-out sensitivity analysis for ‘FN-BMD’ on ‘IVDD’ [file 223_2023_1165_MOESM4_ESM.pdf]

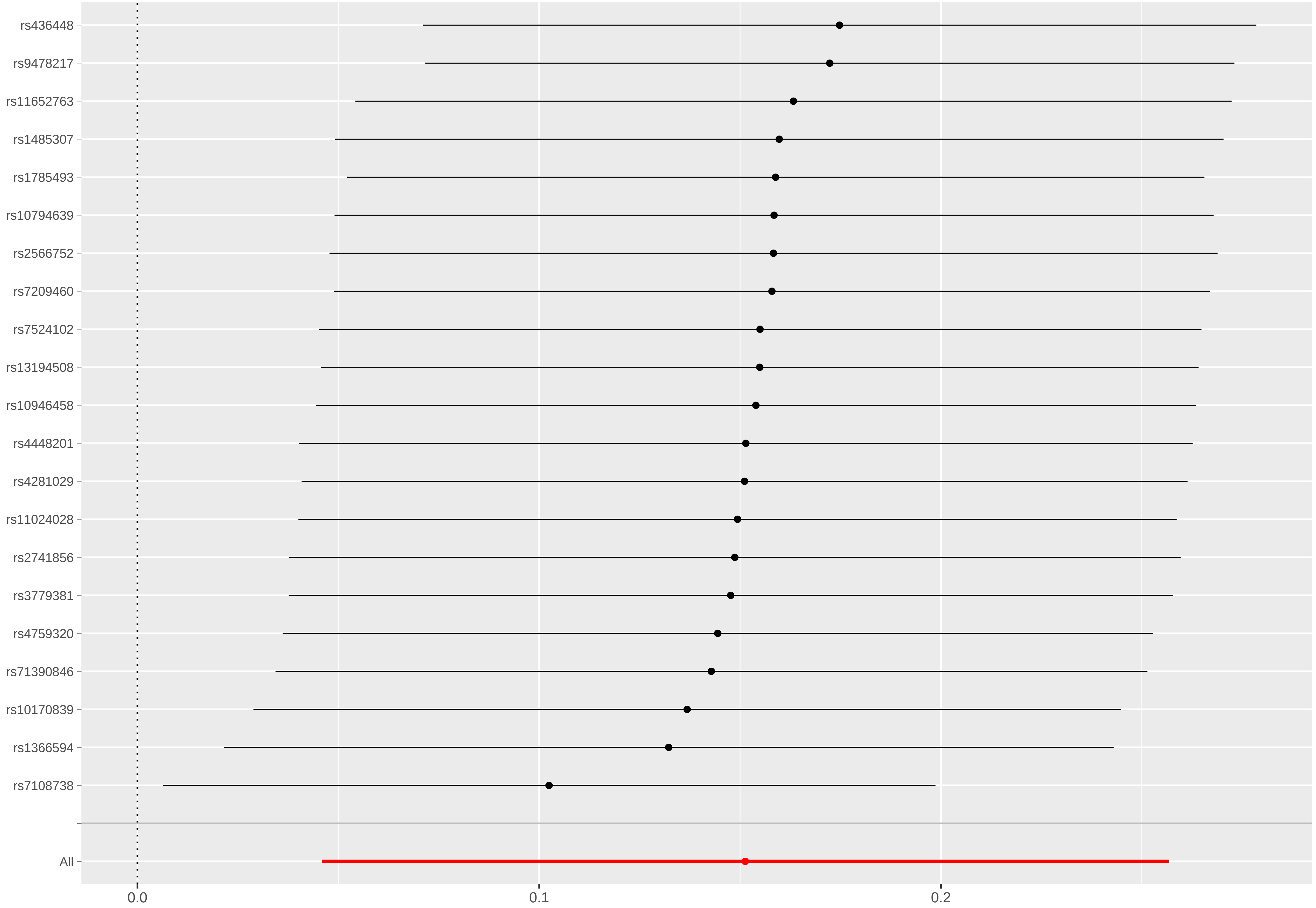

MR leave−one−out sensitivity analysis for
'Femoral neck bone mineral density || id:ieu−a−980' on 'Other intervertebral disc disorders || id:finn−b−M13_INTERVERTEB'
