# Supplementary material for: Intervertebral Disk Degeneration and Bone Mineral Density: A Bidirectional Mendelian Randomization Study
Source: Calcif Tissue Int. 2023 Nov 17;114(3):228–36. doi: 10.1007/s00223-023-01165-1 (PMC10902056; doi:10.1007/s00223-023-01165-1)
Supplement: Supplementary file 5 — Supplementary Figure 5 MR leave-one-out sensitivity analysis for ‘LS-BMD’ on ‘IVDD’ [file 223_2023_1165_MOESM5_ESM.pdf]

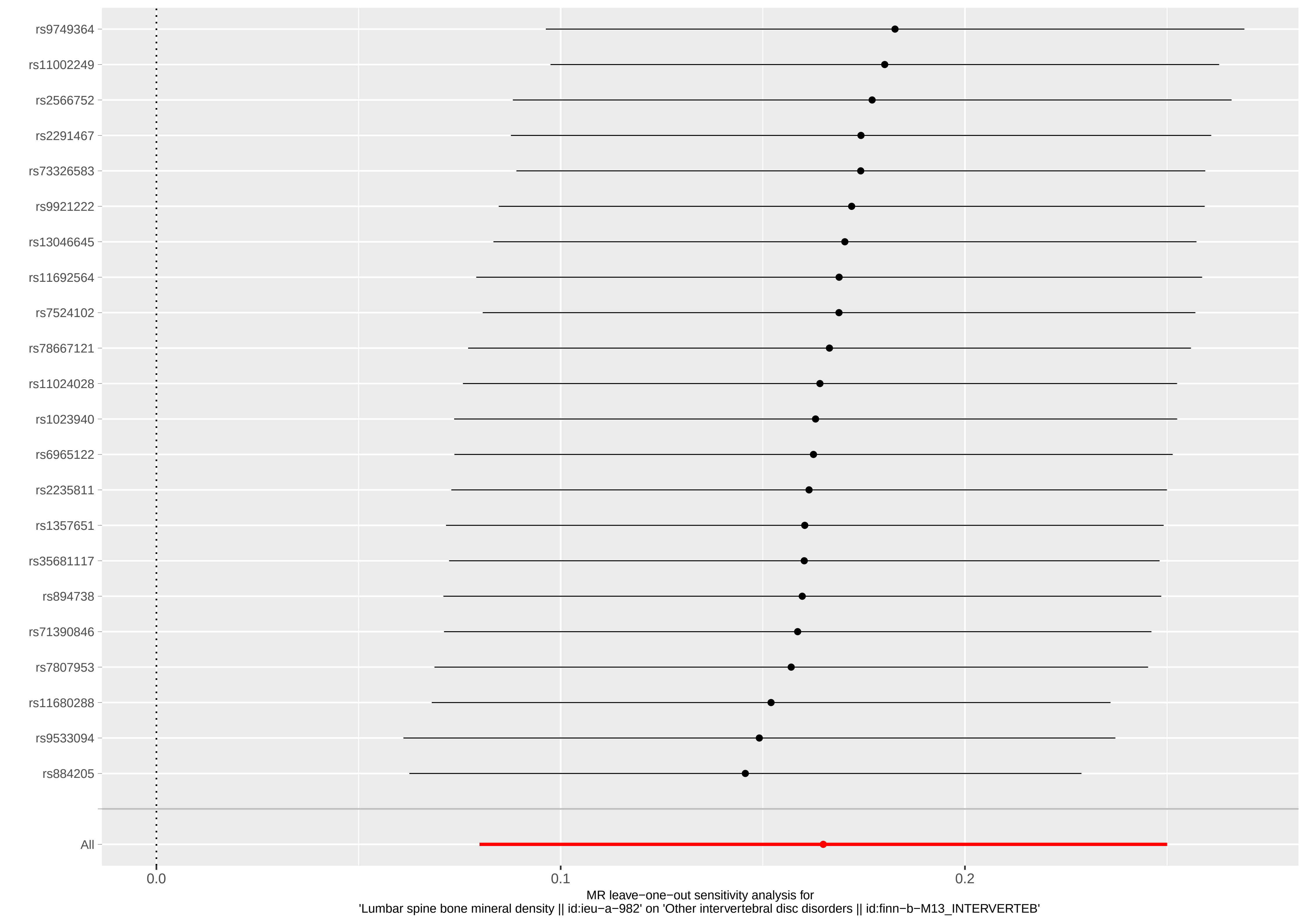

rs9749364
rs11002249
rs2566752
rs2291467
rs73326583
rs9921222
rs13046645
rs11692564
rs7524102
rs78667121
rs11024028
rs1023940
rs6965122
rs2235811
rs1357651
rs35681117
rs894738
rs71390846
rs7807953
rs11680288
rs9533094
rs884205
All
0.0
0.1
0.2
MR leave−one−out sensitivity analysis for
'Lumbar spine bone mineral density || id:ieu−a−982' on 'Other intervertebral disc disorders || id:finn−b−M13_INTERVERTEB'
