# Supplementary material for: Intervertebral Disk Degeneration and Bone Mineral Density: A Bidirectional Mendelian Randomization Study
Source: Calcif Tissue Int. 2023 Nov 17;114(3):228–36. doi: 10.1007/s00223-023-01165-1 (PMC10902056; doi:10.1007/s00223-023-01165-1)
Supplement: Supplementary file 6 — Supplementary Figure 6 MR leave-one-out sensitivity analysis for ‘IVDD’ on ‘heel-BMD’ [file 223_2023_1165_MOESM6_ESM.pdf]

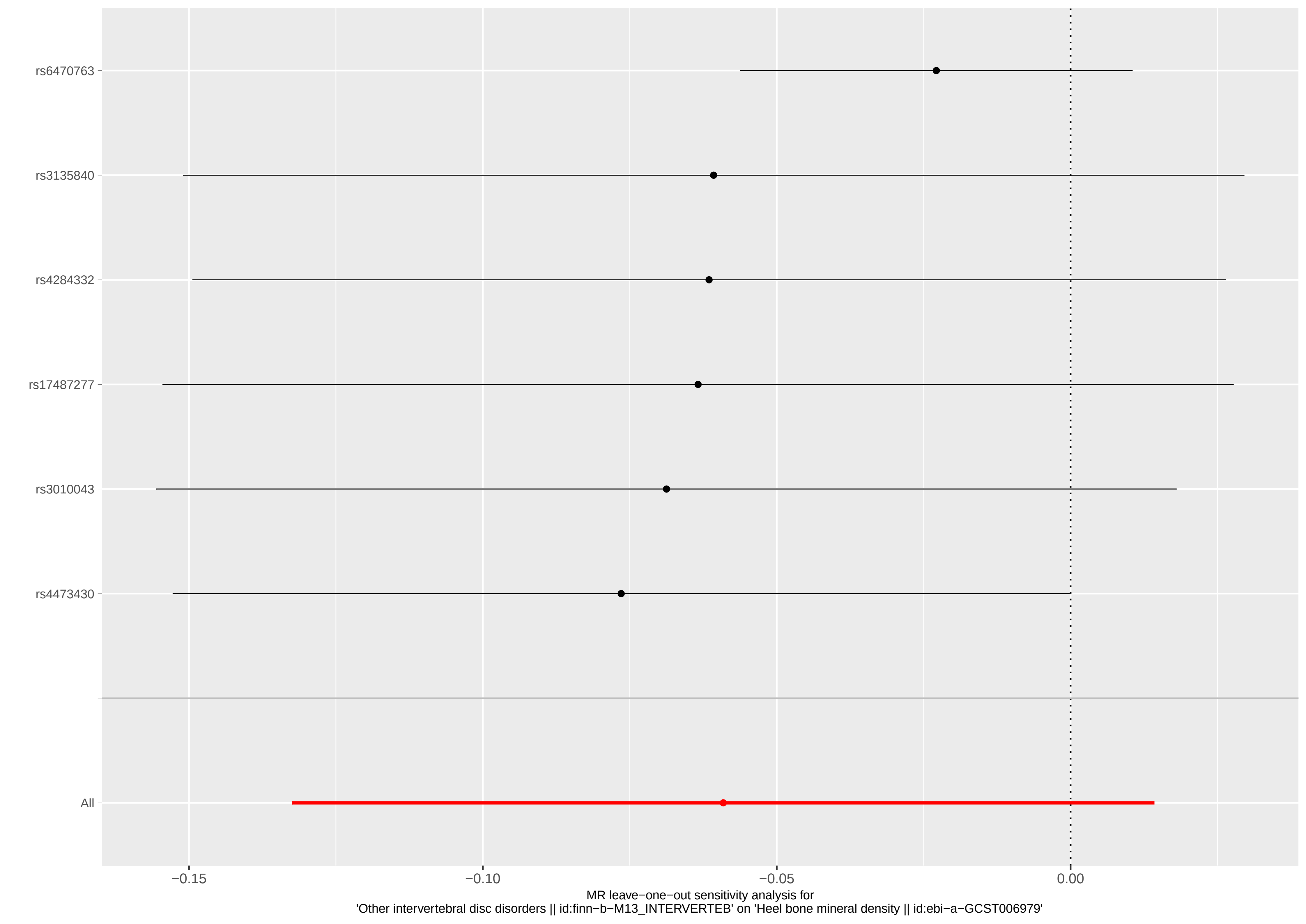
rs6470763
rs3135840
rs4284332
rs17487277
rs3010043
rs4473430
All
−0.15
−0.10
−0.05
0.00
MR leave−one−out sensitivity analysis for
'Other intervertebral disc disorders || id:finn−b−M13_INTERVERTEB' on 'Heel bone mineral density || id:ebi−a−GCST006979'
